# Simultaneous occurrence of subarachnoid hemorrhage and cerebral venous sinus thrombosis: A systematic review of cases

**DOI:** 10.1002/ccr3.6200

**Published:** 2022-08-03

**Authors:** Damilola Jesuyajolu, Olatomiwa Olukoya, Terngu Moti

**Affiliations:** ^1^ Department of Neurosurgery Association of Future African Neurosurgeons Yaounde Cameroon; ^2^ Department of Neurosurgery Surgery Interest Group of Africa Lagos Nigeria

**Keywords:** anticoagulation, cerebral venous sinus thrombosis, CVST, SAH, subarachnoid hemorrhage

## Abstract

Although the leading causes of subarachnoid hemorrhage (SAH) are aneurysm rupture and arteriovenous malformations, cerebral venous sinus thrombosis (CVST) can, in rare cases, be associated with SAH. This phenomenon is an uncommon presentation, with less than a hundred cases reported based on our review of the literature. The purpose of this review is to highlight what is known regarding these cases, how they are managed and to highlight the need for further studies that will serve as a basis for the development of a standard management guideline across board. The following databases were searched: PubMed and Ovid Embase. A complementary search of Google Scholar and AJOL was done. Gray literature search was also conducted on the Google search engine for any additional relevant papers. We were able to extract data regarding 33 cases from 29 identified studies. The mean age was 46.6 ± 14.08. 17 (51.5%) of the cases were female, and the female‐to‐male ratio is 1.1:1. Headache was by far the commonest symptom, occurring in 82% of cases followed by seizures in 42% of cases. Four patients (12%) had loss of consciousness while 5 patients (15%) had some form of focal neurologic deficit. Twenty patients had cerebral venous sinus thrombosis in at least two different sinuses. The superior sagittal sinus was the most common location for CVSTs (79%), followed by the transverse sinus (57.5%). Twenty‐nine cases (89%) were managed with anticoagulation alone and one case had a mechanical thrombectomy. We have performed a comprehensive review of cases that had the simultaneous occurrence of SAH and CVST and have identified their peculiarities and the challenges to management. Further research is needed in order to identify a causal relationship and to serve as a basis for the development of a standard management guideline across the board.

## Graphical abstract

Cerebral venous sinus thrombosis and subarachnoid hemorrhage can occur together and may have a causal relationship. Care needs to be taken to diagnose and treat these conditions when they occur together.

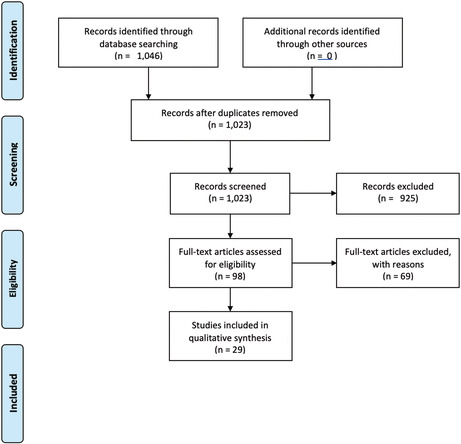

## 1 INTRODUCTION

Although the leading causes of subarachnoid hemorrhage (SAH) are aneurysm rupture and arteriovenous malformations, cerebral venous sinus thrombosis (CVST) can, in rare cases, be associated with SAH.^1^ This phenomenon is an uncommon presentation, with less than a hundred cases reported based on our review of the literature. CVST itself typically presents with headache, nausea, vomiting, weakness, loss of vision, and seizure.^2^ Because of its rarity, a high index of suspicion is important in making the diagnosis.^3^

CVST accounts for 1% of all strokes^4^, ^5^ and has a mortality as high as 30% with the annual incidence ranging from 0.22 to 1.57 per 100,000. It is more common in women than men.^6^, ^7^ Multiple reversible and irreversible factors are associated with CVST and include surgery, thrombophilia, antiphospholipid syndrome, cancer, inflammatory bowel disease, use of the oral contraceptive pill, infection, and pregnancy.^8^

The reason why SAH might occur together with CVST is still debated. In some cases, they could be coincidental. However, there are many hypotheses with regards a causal relationship between the two entities; hence, there is a need to review all the cases in the existing literature to see the similarities and differences across these rare cases and presentations. The purpose of this review is to highlight what is known regarding these cases, how they are managed and to highlight the need for further studies that will serve as a basis for the development of a standard management guideline across board. In this article, we systematically reviewed all such published cases of CVST and SAH occurring concurrently, noting their common characteristics, imaging findings, treatment, and outcomes.

## 2 METHOD AND MATERIALS

### 2.1 Source of information and search

We followed the PRISMA guidelines for conducting systematic reviews. To identify potentially relevant papers, the following databases were searched: PubMed and Ovid Embase. A complementary search of Google Scholar and African Journal Online (AJOL) was done. Gray literature search was also conducted on the Google search engine for any additional relevant papers. The results were exported into an excel document and duplicates were removed. The search strategy is presented in Table A1 in Appendix 1.

### 2.2 Selection criteria

We identified cases where SAH and CVST occurred together. We included case reports and case series, which included the aforementioned. We excluded posters, abstract‐only papers, reviews, meta‐analyses, commentaries, and letters to the editor. We excluded articles that were not written in the English language.

### 2.3 Selection of sources of evidence

**FIGURE 1:**
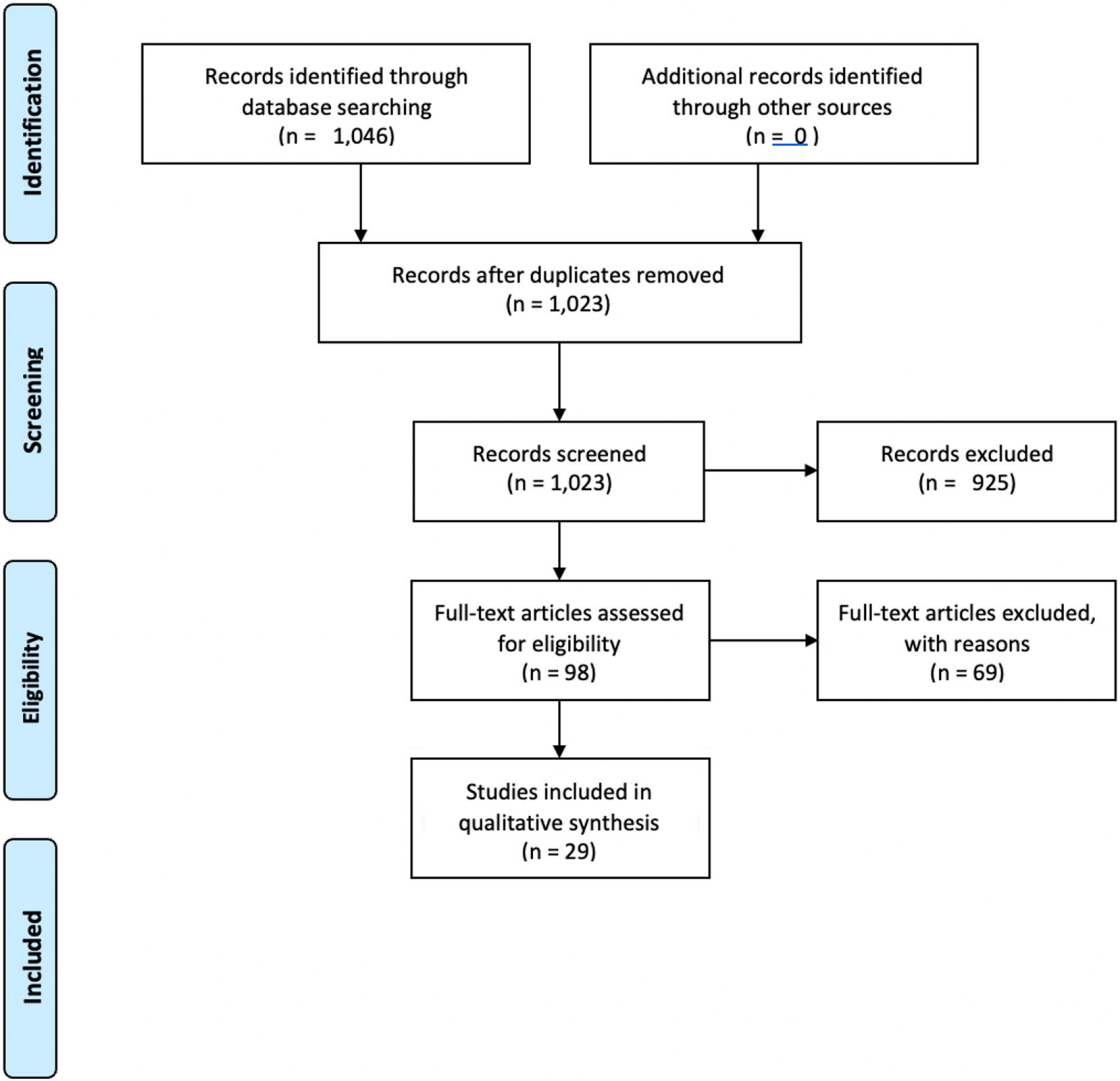
Prisma flow chart

Three reviewers working independently evaluated the titles, abstracts, and then full text of all cases identified by our searches for relevant papers. We resolved disagreements on study selection and data extraction by consensus where necessary. The process is summarized in Figure 1.

### 2.4 Data extraction

A data‐charting form was jointly developed by the three authors to determine which data to extract. The reviewers then read each article extensively and populated the data extraction form with relevant details. The authors continually discussed the results and continuously updated the data‐charting form in an iterative process. We extracted data on the characteristics of each patient/case (Age, gender, symptoms at presentation and their duration), diagnostic modalities for the subarachnoid hemorrhage and CVST, the location of the SAH and CVSTs, possible etiology (presence of aneurysms, trauma, coagulation disorders), the treatment modalities and the outcomes. We were able to extract data regarding 33 cases from 29 identified studies. A summary of the case studies is seen in Table A2 in Appendix 1.

### 2.5 Investigated patient characteristics

The mean age was 46.6 ± 14.08. 17 (51.5%) of the cases were female, and the female‐to‐male ratio was 1.1:1. One case had hyperhomocysteinemia while another had C667T mutations (Heterozygous for methylenetetrahydrofolate reductase). Only one case had a recent use of antiplatelets (Clopidogrel) prior to the diagnosis of CSVT and SAH.

### 2.6 Clinical symptoms and Imaging

Headache was by far the commonest symptom, occurring in 82% of cases followed by seizures in 42% of cases. Four patients (12%) had loss of consciousness while 5 patients (15%) had some form of focal neurologic deficit. Other symptoms included dizziness, nausea, vomiting, gait disturbance and ataxia. The diagnostic modalities used included a non‐contrast computed tomography (CT) scan, computed tomography angiography (CTA), computed tomography venography (CTV), magnetic resonance imaging (MRI), magnetic resonance venography (MRV), and digital subtraction angiography (DSA). Twenty‐five (75.7%) of the studies had an MRI done, while only 17 (51.5%) of them had a further MRV done to confirm the venous thrombosis. Twenty‐seven of the cases had a non‐contrast CT scan done which showed evidence of subarachnoid hemorrhage. Four patients later had a CTA, while another three had a magnetic resonance angiography (MRA) done to exclude an aneurysmal cause. Only one patient had a CTV done.

### 2.7 Diagnosis, treatment, and outcomes

Twenty patients (60.6%) had cerebral venous sinus thrombosis in at least two different sinuses. Of the different locations for the CVSTs, the superior sagittal sinus was the most common location (79%), followed by the transverse sinus (57.5%). Only one case of CVST and SAH had an accompanying intracerebral hemorrhage (ICH). The locations of the subarachnoid hemorrhage were more diverse, ranging from the perimesencephalic areas and cerebral convexities to the Sylvian fissures and interhemispheric fissures. Twenty‐five of them involved the cerebral convexities while 7 of them involved the subarachnoid cisterns. Most of the subarachnoid hemorrhages were non‐aneurysmal. Only one patient had an aneurysmal rupture; the location of the aneurysm was in the anterior communicating artery. Twenty‐nine cases (89%) were managed with anticoagulation alone and one case had a mechanical thrombectomy first prior to anticoagulation. One case was managed with dehydration, scavenging free radicals, and nerve protective therapy, while another was managed with hydration and osmotic diuresis (with an eventual decompressive craniectomy for persistent raised ICP). The only case of the aneurysm was managed with coil embolization. All 28 cases that discussed the status at discharge and a few weeks after reported different ranges of improvement; recovery ranged from improvement in symptoms and partial recanalization to full recovery and full recanalization of the vessels.

## 3 DISCUSSION

Subarachnoid hemorrhage should be considered in the event of a sudden worsening headache.^9^ The diagnostic modality of choice for subarachnoid hemorrhage in the initial stages is a non‐contrast CT scan.^9^, ^10^ With an equivocal result, a lumbar puncture is advised; however, given the increased sensitivity of a non‐contrast CT scan within the six‐hour timeframe, the choice to undertake a lumbar puncture should be through a shared decision‐making process.^9^ As aneurysms are a common cause of SAH, CTA scans are important in demonstrating a causative aneurysm.^9^ A non‐contrast CT scan is also a useful diagnostic modality for diagnosing a CVST as it can show findings that include, but are not limited to, venous sinus or deep vein hyper‐density.^11^, ^12^ CT venography and/or MR venography are recommended diagnostic modalities of choice, as recommended by the European Stroke Organization.^13^

Transverse sinuses, superior sagittal sinuses, and the sigmoid sinus are the most common sites of CVSTs, and in most cases, multiple sinuses are affected.^14^, ^15^, ^16^ This is consistent with the findings of our review study, which showed that multiple sinuses were frequently affected with the superior sagittal sinus and the transverse sinus being the commonest sites. In cases with a coexisting cerebral venous sinus thrombosis, the perimesencephalic region is a common location for non‐aneurysmal SAH.^17^, ^18^ The most common locations for aneurysms are in the circle of Willis, particularly the anterior communicating artery and the internal carotid artery.^19^, ^20^

A third of CVST cases might present with intracerebral hemorrhage.^21^ However, there is a paucity of literature on how common it is for CVST to occur with subarachnoid hemorrhage. One hypothesis of ‌why SAH may occur simultaneously with CVST is that the blood from the ensuing hemorrhagic infarct (resulting from the venous thrombosis) may extend into the subarachnoid space.^22^ This may certainly be the case in some of our findings where there was parenchymal hemorrhage besides the presence of CVST and SAH; however, its absence (also seen in many of the cases identified) may suggest a more direct causal relationship. A leading hypothesis supporting this stipulates that when CVST occurs, the ensuing secondary venous hypertension could be transmitted to the cortical veins, leading to the dilation and rupture of the fragile thin‐walled cortical veins in the subarachnoid space.^17^, ^23^ Sometimes, the occurrence of both entities together could be coincidental, as seen in instances with an aneurysmal cause of the SAH, like in some of the identified cases.

Regardless of etiology, the treatment of a patient with SAH and CVST occurring concurrently can pose a dilemma. The risk of rebleeding in SAH is high, and in the absence of immediate surgical intervention for aneurysmal SAH, antifibrinolytics have been advised.^24^ This is in sharp contrast to the standard treatment of CVSTs, which involves rapid anticoagulation and the stoppage of any prothrombotic medications.^11^ Using systemic anticoagulation where simultaneous subarachnoid hemorrhage exists might worsen the hemorrhage. In cases of simultaneous CVST and ICH, endovascular interventional therapy has been ‌ beneficial.^25^ There is a possibility that such interventions may also yield positive results when used in cases of CVST occurring with SAH. Interestingly, in our study, despite the SAH, most of the patients were treated with anticoagulant therapy with good outcomes reported. Despite our comprehensive review, this study was not without limitations. Because we excluded articles that were not in the English Language, we could have potentially missed relevant literature. There was also heterogeneity in the way the case reports were reported which meant some relevant data could have been missed. Regardless, this review will contribute to the growing body of work on this occurrence.

## 4 CONCLUSION

We have performed a comprehensive review of cases that had the simultaneous occurrence of SAH and CVST and have identified their peculiarities and the challenges to management. Further research is needed in order to identify a causal relationship and to serve as a basis for the development of a standard management guideline across the board.

## AUTHOR CONTRIBUTIONS

D.J involved in conceptualization, methodology, software, validation, formal analysis, investigation, resources, data collection, data curation, writing—original draft, writing—review and editing, project administration, supervision, submission, and correspondence. O.O and T.M involved in conceptualization, methodology, software, validation, formal analysis, investigation, resources, data collection, data curation, writing—original draft, writing—review and editing.

## CONFLICT OF INTEREST

The authors hereby declare that there are no competing interests.

## CONSENT

As this was a review of cases in already literature, no individual consent was required. Consent was however obtained by the individual case reports used in this review in accordance with the journal's patient consent policy.

## Data Availability

The data that support the findings of this study are available from the corresponding author upon reasonable request.

## APPENDIX 1

**TABLE A1:** Search strategy

MeSH/Keywords	Database	Time span	Hits
(“subarachnoid haemorrhage”[All Fields] OR “subarachnoid hemorrhage”[MeSH Terms] OR (“subarachnoid”[All Fields] AND “hemorrhage”[All Fields]) OR “subarachnoid hemorrhage”[All Fields]) AND ((“cerebrally”[All Fields] OR “cerebrum”[MeSH Terms] OR “cerebrum”[All Fields] OR “cerebral”[All Fields] OR “brain”[MeSH Terms] OR “brain”[All Fields]) AND (“venous thrombosis”[MeSH Terms] OR (“venous”[All Fields] AND “thrombosis”[All Fields]) OR “venous thrombosis”[All Fields]))	PubMed	Inception ‐ April 07	381
cerebral venous sinus thrombosis.mp. or cerebral sinus thrombosis/ AND subarachnoid hemorrhage.mp. or subarachnoid hemorrhage/	OVID Embase	1974 to 2022 April 07	665

**TABLE A2:** The summary of studies

Case	Author (Year)	Age/Sex	Symptoms (duration)	Imaging	Location of Subarachnoid hemorrhage	Location of CVST	Anticoagulant use/Hypercoagulability	Treatment	Outcome
1	Gajurel BP et al (2021)	58F	Holocephalic headache with seizure on presentation (3 days)	CTH, MRV, CTA	Insular, perimesencephalic, ambient and suprasellar cisterns,	Left Transverse Sinus	None/NT	LMWH, Levetiracetam and 3% Hypertonic Saline	Home on dabigatran 150 mg twice daily; stable on follow‐up
2	Syed K et al (2021)	48 M	Seizure and Altered Mental State (ND)	CTH, MRI, DSA	Bifrontal	Superior Sagittal Sinus	None/ND	IV esmolol 50 mcg/kg/min infusion and nimodipine 60 mg Q4 hourly; levetiracetam 500 mg, then intravenous heparin and later transitioned to coumadin after CVT dx	Treated and discharged with no neurological sequelae
3	Kumar H et al (2021)	25F	SOH, Confusion, LOC (2 days)	MRI, MRV	ND (review image)	Right Transverse Sinus	None/−ve	Half dosage LMWH (60 mg subcutaneously once a day); Then full dose; Then warfarin	Thrombosis resolved within 6 weeks
4	Sun J et al (2018)	57F	Dizziness; N&V (ND)	CTH, MRA, MRV	Partial gutter of the right frontal, parietal, and occipital lobes	Superior and Inferior sagittal sinus	None/−ve	Dehydration, scavenging free radicals, and nerve protection therapy	18‐month follow‐up: no recurrent thrombosis, improvement of non‐fluent aphasia, right limb muscle strength was slightly worse than normal.
5	Amer RR et al (2018)	44F	SOH (ND)	CTA, MRA, MRv	Prepontine cistern	Left Transverse and sigmoid sinus	None/−ve	LMWH (Nadroparin, 6150AXaIU, subcutaneous injection, twice daily) in combination with intravenous warfarin 3 mg/day	ND
6	Abbas A et al (2018)	58 M	LOC secondary to diarrhea and vomiting (ND)	CTH, CTV	Left temporal and parietal lobes	Superior sagittal sinus and straight sinus	None/−ve	LMWH and Rivaroxoban	ND
7	Liu Y et al (2017)	35F	LOC and Seizures (5 days)	CTH, CTA, DSA	Right temporal lobe	Superior sagittal sinus and bilateral transverse sinus	None/ND	Mechanical thrombectomy; Catheter with Urokinase infiltration; LMWH, then anticoagulant	ND
8	Fu FW et al (2017)	45 M	Occipital headaches, N&V (6 hours)	CTH, CTA, DSA, MRI	Perimesencephalic and prepontine cisterns	Right Transverse Sinus	None/−ve	LMWH (Nadroparin, 6150AXaIU, subcutaneous injection, twice daily), warfarin 14 days later	Improvement of symptoms 10 days post‐treatment, complete absorption of hemorrhage at day 16, no neurologic deficit at 3 weeks
9	Uniyal R et al (2017)	38 M	Holocranial headache; Vomiting; R arm weakness (ND)	CTH, MRI, MRA, MRV	Left central sulcus	Superior sagittal sinus	None/ND	LMWH followed by oral anticoagulation	Recovered completely in 7–10 days
10	Unal Ay et al (2016)	78 M	Sudden‐onset thunderclap headache, Stroke (ND)	CTH, CTH Contrast, MRV	Suprasellar cistern	Transverse sinus, Bilateral Sigmoid Sinus, Superior Sagittal Sinus	Clopidogrel/−ve	Heparin infusion	Symptom relief in two weeks
11	Neubauer C et al (2016)	63F	Headache (3 weeks)	CT, MRI, CTA	Left MCA/ACA territories	Superior sagittal and right lateral dural sinus	None/−ve	Heparin followed by oral anticoagulation; Coil embolization, and smasmolytic	Six months follow‐up revealed complete aneurysm occlusion and progressive recanalization of CVT
12	Anderson B et al (2015)	42 M	Uncontrolled Jerking; Dysesthesia; LOC (ND)	CT, CTA, MRI, MRV	Right frontotemporal convexities	Superior sagittal sinus, right transverse, and sigmoid sinus	None/−ve	Heparin then coumadin therapy	MRI at 2 weeks ‐ SAH resolved; MRI 4 months ‐ partial recanalization of the dural sinuses
13	Hassan Aet al (2015)	46 M	Headache; Right‐sided Weakness; Focal seizures (3 days)	CTH, MRV, CTA	Paramedian sulci (Bilateral)	Superior sagittal sinus, transverse sinus and sigmoid sinus	None/−ve	Subcutaneous anticoagulation then Oral anticoagulation for 6 months	Headache resolved over 1 week; Neurological symptoms over 4 weeks; Seizure free; Maintained on anti‐epileptic drugs
14	Hassan A et al (2015)	35 M	Headache; Seizure (2 days)	CTH, MRI, MRV	Bilateral, predominantly over the left frontoparietal sulci	Superior Sagittal Sinus	None/−ve	Dose‐adjusted intravenous anticoagulation and then Oral anticoagulation	Symptoms improved; Lost to follow‐up at 4 months
15	Arévalo‐Lorido et al (2015)	70 M	Progressive Occipital Headache and nausea (ND)	CTH, MRI, MRA	Right Parietal region	Right Transverse Sinus	None/Heterozygote for methylenetetrahydrofolate reductase C667T mutations; rest negative	60 mg LMH to 120 mg and then oral Anticoagulation	ND
16	Sahin N et al (2014)	48F	Headache; gait disturbance (7 days)	MRI, MRV	sulci of the bilateral frontoparietal convexity	Superior sagittal sinus	None/ND	Anticoagulation	Improved
17	Yamamoto et al (2013)	32F	Head dullness; 9 days later headache and then seizure (ND)	CTH, MRI	Basal cisterns, bilateral sylvian fissures, and anterior interhemispheric fissure	Superior sagittal sinus, straight sinus, right transverse sinus	None/−ve	Hydration	Discharged with only a slight visual field defect in the right eye and returned to her previous occupation
18	Sayadnasiri M et al (2012)	42F	Headache, Vomiting, FND, Seizures (3 days)	CTH, MRI, MRV	Right parietal area	Cerebral Dural Sinuses	None/−ve	Anticoagulation	ND
19	Sayadnasiri M et al (2012)	36 M	Headache, FND, Seizures (3 weeks)	CTH, MRI, MRV	Right Sylvian fissure	Right lateral and sigmoid sinuses and also superior sagittal sinus	ND/−ve	Anticoagulation	Discharged with partial recovery 2 weeks later.
20	Kato et al (2010)	52F	Progressive occipital headache, nausea, and vomiting; generalized seizures 4 days into admission (ND)	Cerebral Angiography, CTH	Right temporal sulcus and bilateral cerebellar sulci	Superior sagittal sinus, straight sinus, transverse sinus, and right sigmoid sinus	None/−ve	IV heparin and then Warfarin	Near‐complete neurological recovery within a month.
21	Sharma et al (2010)	59 M	Rapidly progressive and pulsatile headache then generalized seizures (ND)	MRI, MRV	right parasagittal high frontoparietal areas	Superior sagittal sinus and bilateral transverse sinus	None/−ve	Subcutaneous LMWH and then oral warfarin	Clinically satisfactory within six weeks
22	Lai NK et al (2008)	34 M	Headache, Seizure (3 days)	CTH, DSA	Right frontoparietal sulci	Superior sagittal sinus	None/−ve	Intravenous heparin, then warfarin, and anticonvulsants	MRI at 3 months demonstrated partial recanalization of the superior sagittal sinus.
23	Jaiser et al (2008)	53F	Spontaneous, sudden‐onset occipital headache + mild neck stiffness (3 days)	CTH, CTA, MRV	Left frontal	Superior sagittal sinus	None/−ve	IV unfractionated heparin, then warfarin under LMWH cover	Recanalization of superior sagittal sinus after 6 months
24	Ko YP et al (2007)	25F	Headache, FND (3 days)	CTH, MRI, MRV	Left frontal	Superior sagittal sinus	None/−ve	LMWH, then warfarin	Symptoms improved within a few days
25	Lin et al (2006)	44 M	1 episode of focal motor seizure of left arm; Thunderclap headache (ND)	CTH, CTA, MRI	Right parietal sulci	Superior sagittal sinus, transverse sinus	None/−ve	IV heparin and then Warfarin	L arm weakness resolved
26	Rice H et al (2006)	56F	Headache, neck stiffness, photophobia (ND)	MRI, MRV	Several cortical sulci along the right frontoparietal convexity	SSS, left transverse sinus, Dural sinus	None/ND	Anticoagulation	Clinical improvement within 1 week; Radiological improvement at 3 months
27	Shukla et al (2006)	40 M	Sudden headache associated with recurrent vomiting, photophobia and phonophobia + right focal seizures followed by R hemiparesis (ND)	CTH, DSA, MRV	Left sylvian fissure	Superior sagittal, transverse sinuses, inferior sagittal sinus	None/−ve	LMWH, then warfarin	Symptom‐free at discharge
28	Adaletli I et al (2005)	14 M	Headache, nausea, vomiting, diplopia, and gait disturbance (ND)	CTH, DSA	Basal cisterns, bilateral sylvian fissures, anterior hemispheric fissure	SSS, galenic vein, straight sinus	None/ND	Anticoagulation	Symptoms and signs completely resolved
29	Oppenheim et al (2005)	69 M	Sudden‐onset headache (ND)	MRI + DSA	subarachnoid spaces of the right frontal convexity	Transverse and sagittal sinuses	Previous history of DVT	IV Heparin	Complete radiologic and clinical recovery at follow‐up
30	Oppenheim et al (2005)	55F	Headache, neck stiffness, nausea, seizure (ND)	CTH, MRI, DSA	bihemispheric; predominantly in the left insular sulci	sagittal and the left transverse sinus	None/−ve	LMWH	rapid clinical improvement and partial recanalization of the thrombosed sinus
31	Oppenheim et al (2005)	32F	partial seizure that was secondarily generalized. Headaches with a sudden‐onset with vomiting (3 weeks)	CTH, MRI, DSA	diffuse SAH predominating in the anterior interhemispheric sulci	sagittal and right transverse venous sinuses	hyperhomocystinemia	IV Heparin	complete regression of her neurologic signs and headaches
32	Oppenheim et al (2005)	51F	severe headaches associated with focal neurologic symptoms (5 weeks)	CTH, MRI	diffuse bilateral acute SAH, sparing the basal cisterns	Superior sagittal sinus	prophylactic anticoagulant (not specified) for 2 months post ankle surgery	Anticoagulation	Rapid clinical and radiologic improvement
33	Sztajzel et al (2001)	58F	Severe headache of sudden‐onset (1 day)	CTH, CTA, MRI, MRA	Right cerebellar region	Right lateral sinus; right transverse/sigmoid sinus	Previous history of DVT	ND	Resolution of symptoms after 4 weeks
